# Semantic Health Knowledge Graph: Semantic Integration of Heterogeneous Medical Knowledge and Services

**DOI:** 10.1155/2017/2858423

**Published:** 2017-02-12

**Authors:** Longxiang Shi, Shijian Li, Xiaoran Yang, Jiaheng Qi, Gang Pan, Binbin Zhou

**Affiliations:** College of Computer Science and Technology, Zhejiang University, Hangzhou 310027, China

## Abstract

With the explosion of healthcare information, there has been a tremendous amount of heterogeneous textual medical knowledge (TMK), which plays an essential role in healthcare information systems. Existing works for integrating and utilizing the TMK mainly focus on straightforward connections establishment and pay less attention to make computers interpret and retrieve knowledge correctly and quickly. In this paper, we explore a novel model to organize and integrate the TMK into conceptual graphs. We then employ a framework to automatically retrieve knowledge in knowledge graphs with a high precision. In order to perform reasonable inference on knowledge graphs, we propose a contextual inference pruning algorithm to achieve efficient chain inference. Our algorithm achieves a better inference result with precision and recall of 92% and 96%, respectively, which can avoid most of the meaningless inferences. In addition, we implement two prototypes and provide services, and the results show our approach is practical and effective.

## 1. Introduction

As an indispensable part of today's healthcare information systems (HIS), textual medical knowledge (TMK) plays a pivotal role in healthcare knowledge delivery and decision support to both patients and medical practitioners [1, 2]. In recent years, there has emerged a tremendous amount of TMK, which is aroused by continuous digitalization of medical literature, ongoing expansion of biomedical knowledge, and rapid proliferation of hierarchical online healthcare providers. Facing such tremendous amount of heterogeneous TMK, it has become a challenge to organize and integrate relevant information, and then provide useful processed information to users with an efficient approach. In order to deal with the proliferation of TMK, a computation framework should meet the following three basic requirements:

1. The framework should be capable of organizing and integrating heterogeneous TMK and be capable of fusing them with health data from HIS as well, so that it can facilitate knowledge delivery from data to knowledge.
2. The knowledge representation of the framework should support both human and machine interpretable, so that it can support efficient querying and reasoning over vast knowledge contents.
3. The framework should possess a knowledge retrieval function, which is able to automatically update TMK to push the latest knowledge to users.

Unfortunately, existing works in integrating and utilizing the TMK are unable to meet all the above requirements. Most conventional methods utilize heterogeneous knowledge by matching the keywords [3–7]. Computation systems cannot interpret human knowledge and serve inefficiently when performing complex queries such as acquiring syntactic, semantic, and structural information behind the vast TMK. Their knowledge bases are always manually managed and updated, thus are unable to cope with the proliferation of TMK [5, 6, 8–10]. Therefore, an efficient TMK integrating and delivering method is imperative.

As an evolving extension of the World Wide Web, semantic web technologies have shown great potential in integrating and searching the numerous heterogeneous web content. Through organizing the web content into conceptual graphs using ontologies and Resource Description Framework (RDF), semantic web technologies make it possible for the web to “understand” the human knowledge and provide an efficient querying and reasoning framework for the vast heterogeneous web contents. Moreover, the advent of Machine Learning enables the automated construction of large graph knowledge bases. Google's Knowledge Graph, DBPedia, and YAGO are prominent examples [11]. These characteristics of semantic web techniques make it an ideal choice to meet the above requirements when dealing with the tremendous heterogeneous TMK.

In this paper we propose a novel approach to organize and integrate the TMK into conceptual graphs. More specifically, our contributions are as follows:

1. We propose a model to integrate the heterogeneous textual medical knowledge with health data, which can support semantic querying and reasoning.
2. Based on the model, we employ an automatic knowledge retrieval framework to transform the textual knowledge into machine-readable format, so that we construct a Semantic Health Knowledge Graph.
3. We propose an algorithm to prune the meaningless inference over the knowledge graph. Experiment results prove our algorithm improves the performance of inference results.

We then implement the Semantic Health Knowledge Graph utilizing the semantic web techniques and develop two prototypes for semantic querying and reasoning. Our methods can meet the three requirements mentioned above.

The remaining of this paper is organized as follows. We begin by reviewing the related works in Section 2. After describing the problems in Section 3, we introduce our Healthcare Information Organization Model. In the following two sections we describe the knowledge retrieval framework and propose the inference pruning algorithm. In addition, we also implement two prototypes in Section 7. Finally, we discuss our work and conclude the paper in Section 8.

## 2. Related Works

In this section, we review the existing literature on TMK integration and utilization. Some researchers and organizations have paid a lot of efforts to integrate and utilize TMK contents, in order to cope with the explosion of heterogeneous TMK. The mostly used approach is to utilize standard medical terminologies to integrate heterogeneous TMK. Through the standard metathesaurus, for example, Unified Medical Language System (UMLS) [8], ICD9/10, and SNOMED CT [9], heterogeneous TMK can be integrated and queried with the utilization of a terminology mapping strategy. These methods have been applied in a variety of fields [3–6], for example, tranSMART [4], MayoExpert [5], most commercial healthcare information systems [6], and various online healthcare providers. Organizing and integrating the medical knowledge into cases, also known as Case-based Reasoning (CBR), is another famous method to integrate the TMK. However, the construction of CBR knowledge bases always needs experts' participation [12]. Those manually integration methods fail to cope with the rapid growing of the medical knowledge.

Some previous works tried to employ data mining approaches to extract relevant information. Nguyen et al. [7] applied a rule-based classification method to provide user-specific information. Stewart [13] utilized semantic content analysis method for relevant contents retrieval. Wright et al. [14] proposed a framework for sharing clinical decision support content using web2.0. These methods can handle the proliferation of TMK. However, their computation systems are unable to interpret human knowledge and are unable to provide comprehensive and complex retrieval results.

Facing this problem, a number of existing studies have proposed computer-interpretable knowledge representation approaches. Large biomedical ontologies, such as Gene ontology, Disease ontology and many other ontologies from Linked Life Data [10], were manually organized to create computer-interpretable representation knowledge, but they mainly focused on molecular level and needed a lot of human efforts. Ernst et al. [15] proposed an automatic approach for large knowledge graph construction for biomedical science, which were unable to integrate with health data. The IBM Watson healthcare system employed cognitive technologies to process information similarly to a human being by understanding natural language and analyzing unstructured healthcare data [16]. However, high computational cost of Watson hindered its ubiquitous application.

Based on the integrated TMK, how to provide relevant knowledge content to user is another important process, that is, the reasoning process. Generally, there are mainly four types of reasoning methods that utilizing the integrated TMK for decision support: Reasoning based on Similarity Matching, Probabilistic Reasoning, Logical-based Reasoning and Reasoning based on Machine Learning. Reasoning based on Similarity Matching is the most used method, which is used in most commercial healthcare information systems [6], CBR systems [17], and so forth. Probabilistic Reasoning and Logic-based Reasoning are widely used in rule-based Clinical Decision Support Systems. Probabilistic Reasoning used Bayesian inference rule to compute conditional probability thus finding the most relevant content, while Logic-based Reasoning uses logical statements or axioms to assist decision making [18]. Reasoning based on Machine Learning uses techniques such as classification and clustering to provide user-relevant content, as used in [7, 13, 15, 16]. However, few reasoning works focus on the validation of inference results. Without validation, inference may encounter inaccurate and meaningless results.

In summary, conventional methods mainly focused on creating connections straightforwardly through keywords matching from multiple heterogeneous knowledge sources. Moreover, computers were unable to explicate human knowledge and performed poor when met complex queries such as acquiring syntactic, semantic and structural information which cannot be obtained from TMK directly. Integrating with health data has been always neglected. Their knowledge bases were always manually managed and updated to the latest knowledge, thus are unable to cope with the proliferation of TMK. In addition, few reasoning works focus on the validation of inference results.

## 3. Problem Description

In this section, we introduce some basic preliminary knowledge, including health data and textual medical knowledge sources. Then the problem of this paper is described.

### 3.1. Preliminary

#### 3.1.1. Health Data Description

**Figure 1:**
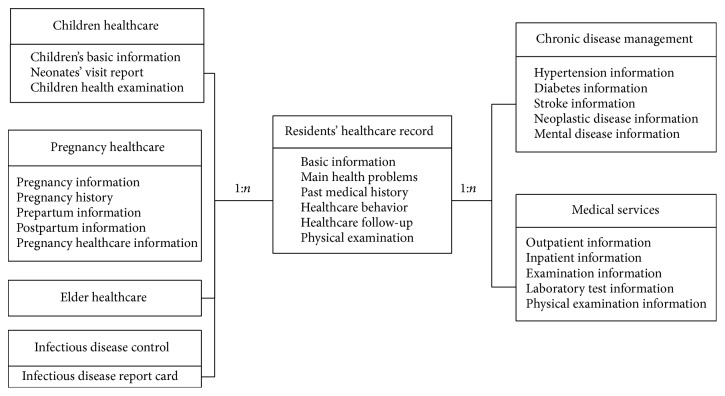
Overall architecture of health information system.

Health data used in this paper were collected from Health Information System of a city in Zhejiang (HISCZ), China. The system was designed for residents' health data integration and sharing through a city-level data sharing platform of the city health bureau. HIS of hospitals, clinics, or other health agencies in the city must comply with the HISCZ data storage standard. Meanwhile, HISCZ also complies with the classification and coding format for value domain of health data element, the national health data sharing standard of China (CHDE) [19]. However, some clinical narratives, such as chief complaint from doctor interviews, are not stipulated in CHDE. Therefore, health data we studied from HISCZ consists of structured, semistructured, and unstructured data. The overall architecture of HISCZ involves six main parts of residents' healthcare records (as shown in Figure 1), including chronic disease management, elder healthcare, children healthcare, pregnant healthcare, disease control, and medical service. Here we mainly focus on medical service data, which contain outpatient and inpatient medical records.

#### 3.1.2. Textual Medical Knowledge Sources

In this paper we study two types of textual medical knowledge sources: open healthcare contents from the web and a medical book [20] which was retrieved by Optical Character Recognition (OCR) technique. The open healthcare contents are mainly about the healthcare materials for layman which contain two parts: Self-Diagnosis of Common Diseases [21] and Merck Diagnostic Manual Chinese Edition [22]. Both of the knowledge sources are arranged in a specific document structures including titles, sections, and listings.

### 3.2. Problem Description

Our goal is to explore an efficient way to organize, integrate, and deliver the heterogeneous tremendous TMK using semantic web technologies. Therefore, there are mainly three challenges:

1. A model is needed to organize and integrate the heterogeneous medical information. Health data from Electronic Health Records (EHRs) systems are always highly complex. It contains a mixture of many continuous variables and a large number of discrete concepts [23]. Most of them are represented as unstructured free-text format that need nature language processing. In addition, healthcare-related terminologies may vary from different doctors [24]. As well as the health data, TMK also faces similar problems, such as multiple heterogeneous variables, unstructured free-text format, and inconsistent terminology usage. Therefore, we need to propose a model to deal with this heterogeneous medical information. Moreover, in order to make computers understand this information, the conceptual graph based knowledge representation methods must be taken into consideration.
2. To automatically retrieve knowledge from heterogeneous textual knowledge sources, effective algorithms are required to process these textual TMK as the model represented.
3. For the delivery of reasonable health knowledge, an inference algorithm is needed when we perform query and inference over the graph knowledge base.

In the following sections we will describe our methodology which are able to overcome those challenges.

## 4. Healthcare Information Organization Model

### 4.1. Model Overview

**Figure 2:**
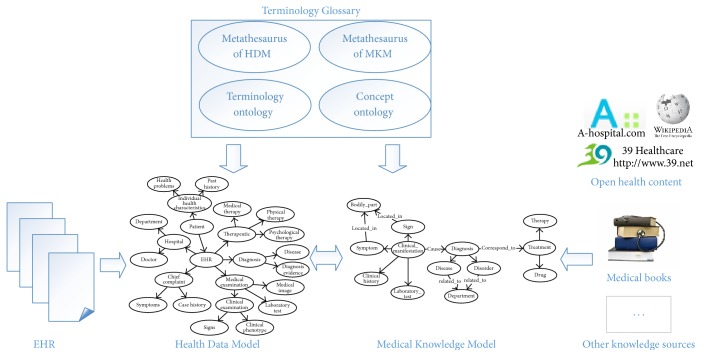
Model overview.

In order to organize and integrate the heterogeneous healthcare information, we propose a Healthcare Information Organization Model to normalize the heterogeneous healthcare information into a sharable and consistent format. To enhance semantic applicability, we model those information using conceptual graph representation. An overview of our model is illustrated in Figure 2. Our model consists of three parts: Medical Knowledge Model (MKM; see Figure 4), Health Data Model (HDM), and Terminology Glossary (TG). Medical Knowledge Model is used to organize the TMK into conceptual graphs. Health Data Model is used to define and normalize the detailed structures and relationships of the complex and unstructured health data from EHRs, thus facilitating integration with TMK. Terminology Glossary provides metathesaurus to express the instances of both TMK and HDM and provides semantic mappings to achieve integration. In the following subsections we will describe each part in detail.

### 4.2. Medical Knowledge Model

**Figure 3:**
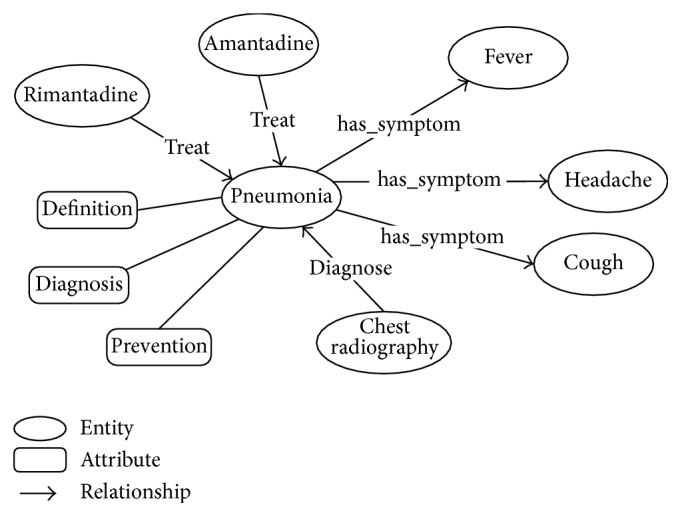
Illustration of the conceptual graph knowledge representation of encyclopedia on pneumonia.

**Figure 4:**
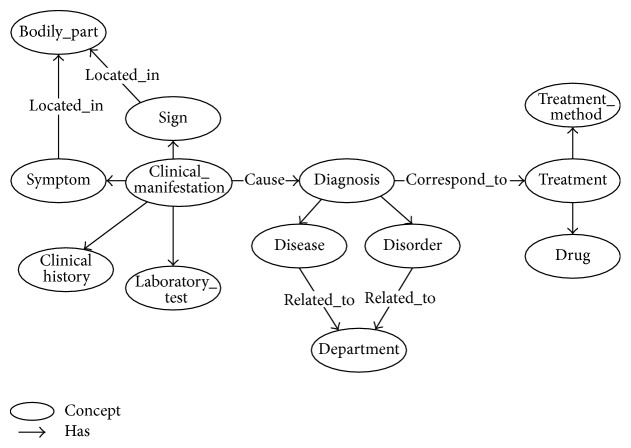
Illustration of Medical Knowledge Model (in part).

Medical Knowledge Model (MKM) is used to define the schema of knowledge to represent the TMK into conceptual graphs and to integrate with health data. In order to enable computers to explicate medical knowledge, we abstracted the textual format medical knowledge into a graph expression based on the conceptual graph knowledge representation [25]: medical terminologies are classified and served as the vertexes (entities) of the graph, and sentences that describe relationships between medical terms are abstracted as the verges of the graph. In addition, the descriptive knowledge which explains the entities is taken as the attributes of the entities. This metaknowledge composes the basis of our graph knowledge base.  Figure 3 illustrates the graph representation of encyclopedia on pneumonia.

Based on the graph knowledge representation, our MKM defines the classes (or concepts) of the medical entities with their relationships of medical knowledge that needed to be abstracted and integrated. Entities of concepts in MKM are defined in the Terminology Glossary. In order to illustrate the complicated semantics and relationships in the knowledge model, we adopt ontology technique to represent the MKM. Actually, there are many existing knowledge models in biomedical domain. Most of those knowledge models focused on a specific domain. For example, the OBO foundry [26] has developed many biomedical ontologies that are both logically well-formed and scientifically accurate. The SemanticHealthNet [27] project also developed several biomedical knowledge models for sharing knowledge. Such knowledge models can be considered and reused to build the MKM. In this paper, we specifically focus on the knowledge in clinical diagnosis and treatment process. Therefore, we build an upper ontology model to describe the concepts and relationships in clinical diagnosis and treatment. The existing domain-specific knowledge models can be integrated through the MKM. To achieve theoretically rationality, we use the existing medical ontologies as reference [28, 29]. Our MKM consists of 3 parts:

1. Clinical manifestation: a representation of a bodily feature of a patient that is recorded by a clinician about an illness [28], such as signs, symptoms, clinical histories, and laboratory tests.
2. Diagnosis: the conclusion of an interpretive process that has as input a clinical picture of a patient and as output an assertion to the effect that the patient has a disease of such and such a type [28], such as a disease or disorder.
3. Treatment: the medical or surgical management of a patient [28], including treatment method and treatment plan.

### 4.3. Health Data Model

**Figure 5:**
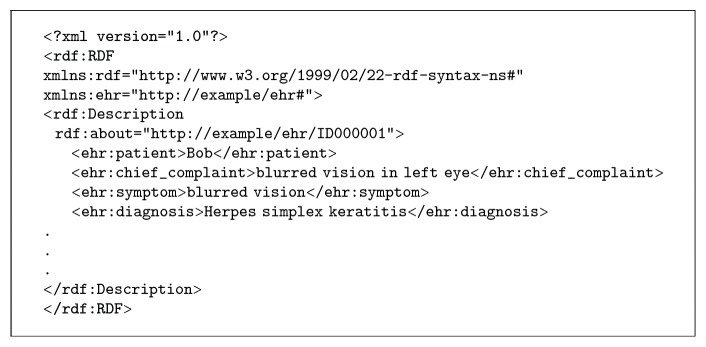
Illustration of RDF representation of EHR.

In order to integrate the heterogeneous health data with medical knowledge, it is necessary to express these data into a sharable and consistent format. Fortunately, numerous studies have noticed this problem. The semantic web provides a common framework that allows data to be shared and reused across applications, enterprises and community boundaries [30], and receives widely adopted in healthcare data integration [31–33]. Moreover, existing standards such as HL7 [34], SNOMED CT [9], and ICD 9/10 have been established to normalize the conceptual model of health data [35]. Hence, we adopt semantic technologies to achieve the integration of health data with medical knowledge. Health Data Model (HDM) is derived from the original data schema and supervises the health data into semantic format, while the data entities are defined in the Terminology Glossary. We use an ontology model to express the HDM. The normalized health data tuples are stored in RDF to integrate with medical knowledge, as illustrated in Figure 5.

Since the health data we retrieved are stored in a relational database from EHR systems, their logical structures are defined using entity-relationship models (ERM) [36]. As a consequence, we transform the ERM to ontological model using the following steps:

1. Identify the health data that need to integrate with knowledge.
2. For the unstructured health data, build the structural ontological model of health data based on the existing standard.
3. After the health data are wholly structuralized, give the detailed definition of the data domain and attributes.

**Figure 6:**
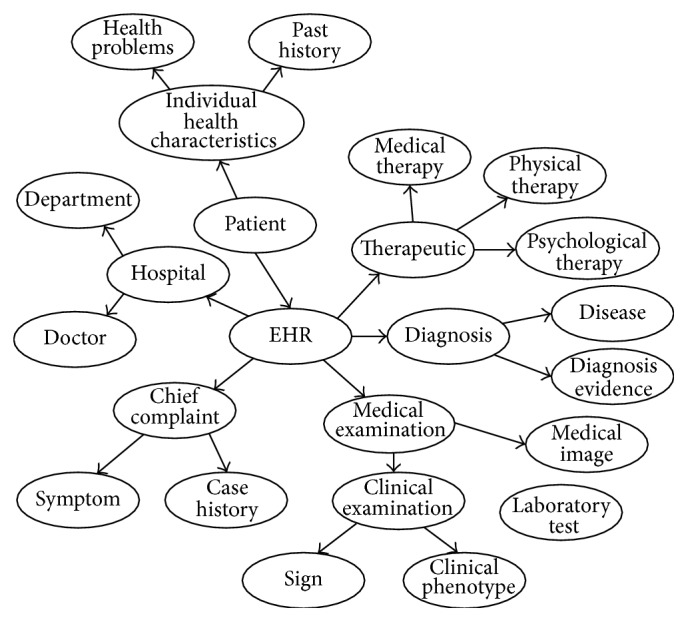
Illustration of Health Data Model (in part).

Based on the above steps, our HDM is depicted in Figure 6.

### 4.4. Terminology Glossary

Terminology Glossary (TG) provides a metathesaurus to express the instances of both health data and medical knowledge and provide semantic mappings to achieve integration. Both MKM and HDM need a metathesaurus to express the concrete instances, such as a fact of medical knowledge or a specific health records. Therefore, the TG contains four parts: a metathesaurus for health data, a metathesaurus for medical knowledge, a terminology mapping ontology between two metathesaurus and a concept mapping ontology of the two models. As illustrated above, the metathesaurus of MKM and HDM can use the existing medical ontologies such as SNOMED CT and ICD. The terminology ontology gives semantic mapping of the words between the metathesaurus used in HDM and MKM, while the concept ontology gives semantic relationships between concepts in HDM and MKM. Through this way we ensure the applicability for different EHR systems. Different EHR systems can share the same knowledge model and only need to modify the TG.

Since our health data comply with the national standard for EHR of China [37] and follow the standard for interface technology of health data sharing and access of Zhejiang Province, our metathesaurus of HDM follows these standards as well. For the metathesaurus of the MKM, we present the detailed information of the Terminology Glossary in Table 1. Due to the lack of authentic standard medical terminology in Chinese [38], some of the terminologies are collected manually from medical books and the open health contents. Since our MKM and HDM share most contents in common, we simply build a mapping ontology between the synonyms of both metathesauruses.

## 5. Automatically Knowledge Retrieval Framework

**Figure 7:**
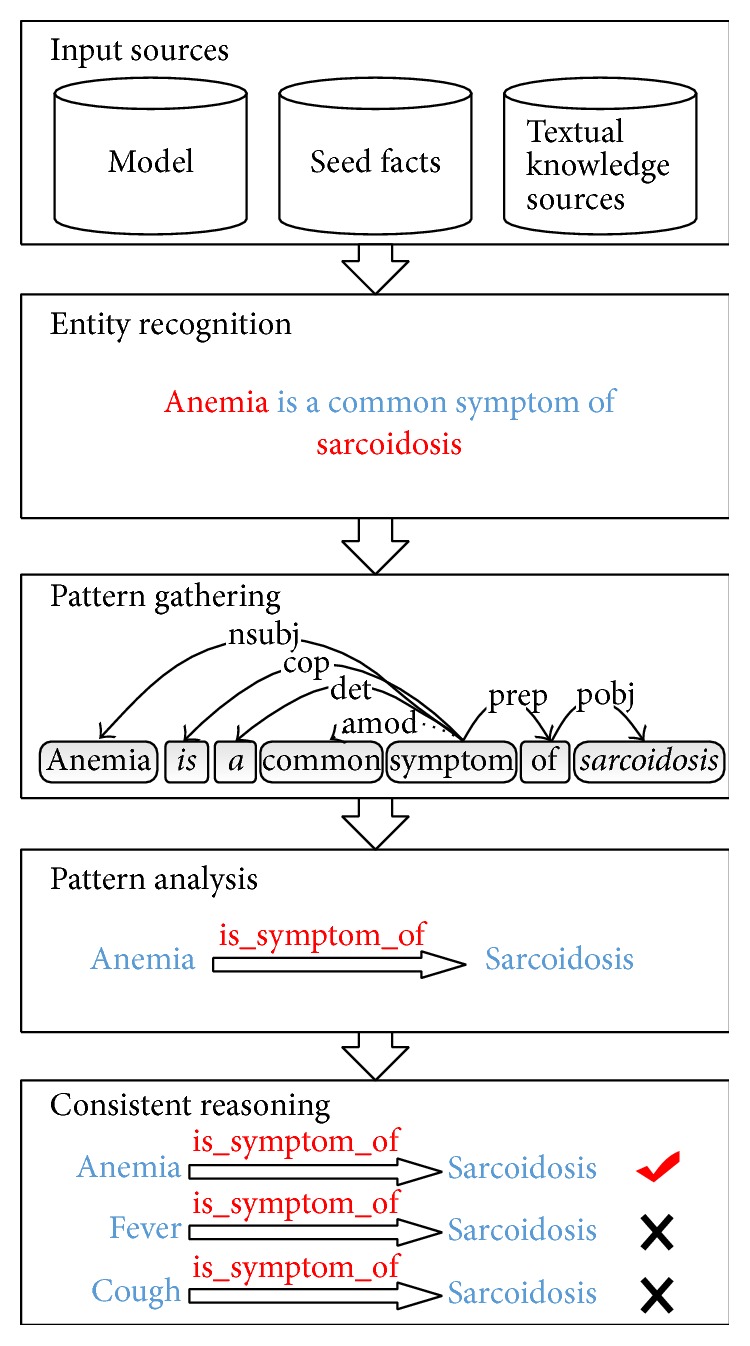
Illustration of facts retrieving framework.

In order to automatically retrieve the healthcare knowledge, we reviewed existing algorithms that used in relations extraction from the web contents [39]. To achieve high precision and recall for medical consideration, we adopt a textual pattern-mining framework used in KnowLife [11, 40] to process the knowledge. We then improve original framework to adapt to the Chinese knowledge sources.  Figure 7 gives an illustration of the facts retrieval framework.

- Input sources: the input of the framework contains 3 parts: a model, seed facts, and preprocessed textual knowledge sources.
  - Model: our model provides the requirements of the facts retrieving framework: MKM provides the relations that need to be retrieved from knowledge sources; TG provides terminology dictionaries for entity recognition. In this paper we mainly consider three types of relationship, depicted in Table 2.
  - Seed facts: seed facts are relations presumed to be true based on expert statements. They are served as basic patterns for facts retrieval. For each relationship we collected seed facts separately, as shown in Table 2.
  - Preprocessed textual knowledge sources: as described above we use two genres of text. The texts are then preprocessed using ICTCLAS [41]. The preprocessed texts are tokenized, split into sentences tagged with parts-of-speech, lemmatized and parsed into syntactic dependency graphs [40], as shown in Table 3.
- Entity recognition: entity recognition procedure identifies the entities occurring in the sentences. A lexical analyzer is required for word segmentation using our dictionary. In this work we use ICTCLAS [41] to perform the entity recognition procedure for Chinese.
- Pattern gathering: pattern gathering extracts the textual patterns from preprocessed knowledge sources. We here extract sentence-level patterns by parsing the syntactical structures of each sentences. The syntactical structures of each sentence were analyzed to find the shortest path in its dependency graph.
- Pattern analyzing: pattern analyzing aims at identifying the most useful seed patterns among all the patterns gathered in the above procedure. We use the Prospera tool [11] to find the salient patterns among the gathered patterns. Based on a frequent item-mining algorithm, Prospera computes the similar patterns and weighted by statically analysis. Seed facts and their cooccurrences with certain patterns served as a basis to compute the confidence. Selected patterns with high confidence above specific thresholds served as candidate patterns for evaluation.
- Consistency reasoning: consistency reasoning aims at pruning the false facts among the facts extracted. We use two methods to deal with the mutual consistency of the fact candidates. Open health knowledge contents are also added for consistency reasoning. We use the Weighted Max Sat Solver in Prospera tool and the crowdsourcing technique. For the crowdsourcing technique, our knowledge base supports the feedback of the users to enable the crowd intelligence thus helping optimize the relationships in the SHKG.

In order to evaluate the results, for each relation we randomly sample 50 retrieved facts and manually verify the facts. For each relation we perform 3 iterations of Prospera. After the retrieval of relations, the textual contents are filled into the attributes of the entities. So far, our Semantic Health Knowledge Graph (SHKG) has already been built. The detailed statistics of our SHKG is shown in Table 4.

## 6. Performing Reasonable Inference over the Semantic Health Knowledge Graph

**Figure 8:**
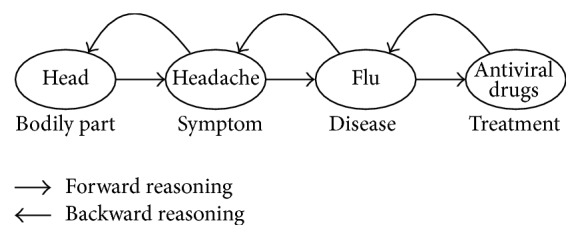
Chain reasoning examples.

After the SHKG construction procedure, we are able to utilize the interconnections between medical terms to perform chain inference rules to explore the complex semantics between entities. In this paper we use first-order predicate logic to perform reasoning on SHKG. Inferences are proceeded by forward chaining and back chaining over the knowledge graph.  Figure 8 shows an example of chain reasoning. Given a specific input in bodily part, we can retrieve the symptoms that are located in this body part and then the possible diseases of the symptoms and corresponding treatments of these diseases and vice versa.

**Figure 9:**
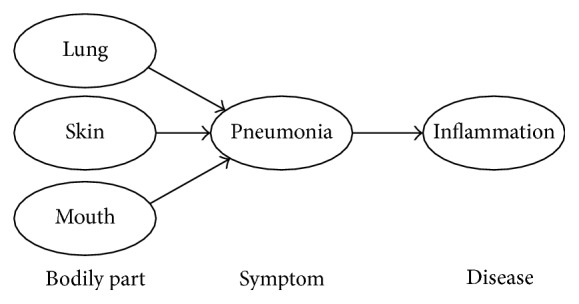
Chain inference example.

Since the SHKG is composed of numerous binary relations between entities, there may encounter some potential problems when performing chain inference rules. Due to the complexity of medical knowledge, SHKG contains numerous relations sharing same precedents or antecedents. Meaningless relation chains would occur when performing chain inference over *n*-to-1 or 1-to-*n* binary relations. For example, as shown in Figure 8, inflammation may occur in bodily parts such as lung, skin or mouth. However, only lung inflammation could cause pneumonia. As a result, only the inference chain (see Figure 9) (lung → inflammation → pneumonia) is reasonable inference while (skin → inflammation → pneumonia) and (mouth → inflammation → pneumonia) both are meaningless inference.

**Figure 10:**
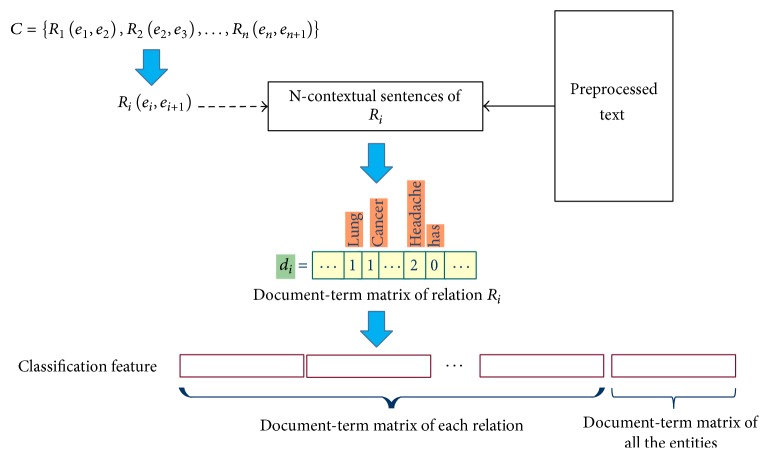
Feature construction procedure of inference chain.

Therefore, it is necessary to prune these meaningless inference results. To formally define the problem, we use *S* representing the whole binary relation set of SHKG. *C* represents the inference chain {*R*_1_(*e*_1_, *e*_2_) → *R*_2_(*e*_2_, *e*_3_) → ⋯→*R*_*n*_(*e*_*n*_, *e*_*n*+1_)} which needs to be revised. The above scenario can be expressed to prune the meaningless inference chain *C*. To find out meaningless inference chain, we can prelabel some inference chains as study materials. Thus, it is a classification problem. Since most medical knowledge is expressed in a context-sensitive grammar, for a specific relation *R*(*e*_1_, *e*_2_) the entities *e*_0_, *e*_2_ from its precedent relation *R*_*p*_(*e*_0_, *e*_1_) and antecedent relation *R*_*a*_(*e*_1_, *e*_0_) can mostly be found in the context around the sentences expressed *R*(*e*_1_, *e*_2_). Hence, we go back to the sentences that relation *R*(*e*_1_, *e*_2_) was retrieved to extract classification features. For each relation *R*(*e*_0_, *e*_1_) from inference chain *C*, the original sentences that include *R* along with the precedent N sentences and the antecedent N sentences are obtained as “N-contextual sentences (N-CS).” To acquire semantic information of the N-CS of *R*(*e*_0_, *e*_1_), we then represent the N-CS in vector space model [42]. For each relation *R* from *C*, the document-term matrix of N-CS of relation *R* is obtained as features. In addition, the document-term matrix of entities in each relation is also added as feature. The detailed feature construction procedure is shown in Figure 10. After the feature construction procedure, classification methods can be used to identify the meaningless inference.

We evaluate our feature construction method using classification methods over 3-chain inferences on our knowledge graph. We manually label 200 3-chain inferences as the study inference set, including 100 meaningful inferences and 100 meaningless inferences. To ensure the effectiveness of evaluation, the 3-chain inferences containing wrong binary relation are excluded. Either the meaningless or reasonable inference chains are manually checked the correctness. The contextual sentence range number is set to 3. We then use Naive Bayes, Logistic Regression, Support Vector Machine, and ID3 Decision Tree to classify these inferences. To ensure robustness, 5-cross validation is performed. The results are shown in Table 5. Among these classification algorithms, Logistic Regression performs the best with both high precision and recall. As a consequence, we use Logistic Regression to prune the meaningless inferences.

## 7. Implementation: Prototypes and Services

Based on the above works, we implement the SHKG using semantic technologies. Two prototypes are implemented to show the semantic applications over the integration of tremendous heterogeneous healthcare knowledge. In this section we will describe the implementation in detail.

### 7.1. Representation of Semantic Health Knowledge Graph

**Figure 11:**
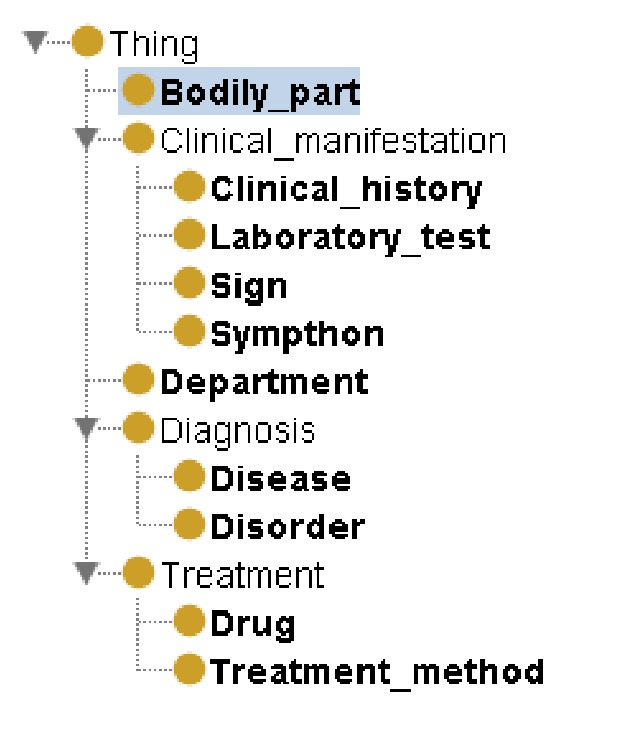
Construction of Medical Knowledge Model using protégé.

In order to represent the proposed model, we adopt semantic web techniques in our work. We use Web Ontology Language (OWL) [43] to describe the ontologies used in our model. OWL is the standard language representing the rich and complex knowledge in semantic web. OWL is also a computational logic-based language, which can provide computer-interpretable reasoning over the represented knowledge. Due to OWL's powerful expressive ability and computation reasoning support, we adopt OWL to represent our model. We then use protégé [44] to create the ontologies of the model.  Figure 11 presents an example of the MKM construction using protégé.

Considering the relations and descriptive knowledge in the SHKG, we use Resource Description Framework (RDF) [45] for representation. RDF is the standard model for data interchange in the semantic web and has features that facilitate semantic applications. Since the health data and medical knowledge are mostly represented using RDF, in order to perform semantic querying and reasoning we use SPARQL to perform semantic querying over health information. SPARQL is a standard semantic query language for RDF and is one of the key technologies of the semantic web. We then use Jena API [17] to implement the framework.

### 7.2. Comprehension of EHRs

As an entrance for personal health, Electronic Health Records (EHRs) have the potential to empower healthcare consumers and improve healthcare [41]. However, most of the EHR contents are made up largely of physician progress notes, discharge summaries and procedure reports, including a lot of professional medical concepts and terminologies [46]. It is hard for patients to understand.

**Figure 12:**
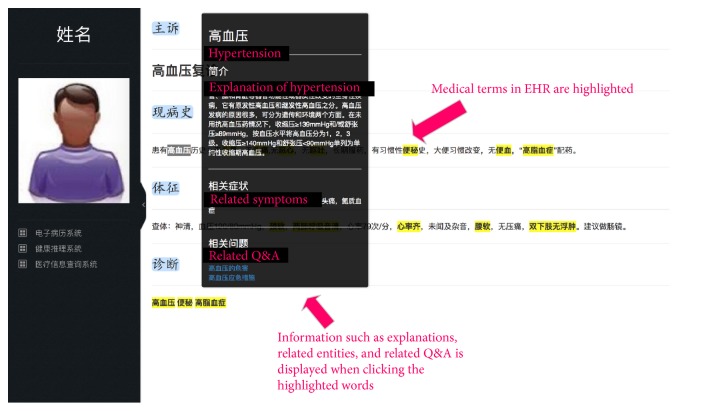
Comprehension of EHRs by the SHKG.

Therefore, we implement an EHR comprehension system based on SHKG, as shown in Figure 12. The semantic integration between heterogeneous knowledge sources and health data makes the health data easily interlinked to multiple knowledge sources. By clicking the highlighted terms of EHRs, the system would display the explanations from the medical books and the related questions from the web. Users can obtain a deeper insight of the health data through clicking the highlighted items from the knowledge. Behind the textual expression of the EHRs, the semantic representation facilitates the query from heterogeneous data to knowledge, not only matching the strings. Based on the computer-interpretable knowledge representation, the system can also provide the most relevant information that are interconnected with items, which illustrates a broader view to the users. The example webpage can be found in http://120.27.128.97/2.html.

### 7.3. Semantic Reasoning over SHKG: A Prototype Service

Based on transforming the textual knowledge into a conceptual graph representation, computers are able to interpret the health knowledge content. In this paper we implement an intelligent diagnose assistant system based on SHKG. The system is available for an interactive use at http://120.27.128.97.

Through automatically integrating the latest knowledge sources such as articles and guidelines, our system can keep pace with the rapidly changing medical researches and translate them to clinical settings. In addition, the integration of health data makes it easy for the delivery of the latest healthcare knowledge.

Given several input symptoms, the system will query the SHKG and provide diagnosis and treatment advices. If the input symptoms are not capable of identifying the disease, the system would ask the users to fulfill the symptoms.

**Figure 13:**
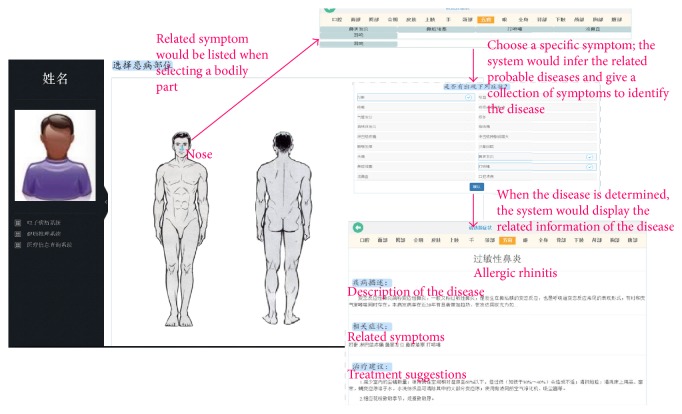
Illustration of intelligent diagnose assistant.

We provide two entrances for users: one is through textual input and the other is through semantic body browser, as shown in Figure 13:

- Textual input box: based on the textual input of symptoms, the system will infer the knowledge base to show the related diseases.
- Semantic body browser: user can simply choose the body part that is related to the symptoms and select symptoms.

In addition, the system will also display the explanations of results and give an integrated illustration from heterogeneous knowledge sources such as medical books and the related questions from the web.

## 8. Conclusion

The tremendous amount of TMK which emerged in recent years provides us with an opportunity to share and utilize these TMK together to explore and get access to valuable useful information. In this paper, we introduce a healthcare information model to organize TMK into conceptual graphs, define consistent data structures for all data involved, and provide semantic mappings between TMK and medical knowledge. And then we optimize a texture pattern-mining framework for automatic healthcare knowledge retrieval and finally consistent reasoning. After that, we propose a contextual inference pruning algorithm to explore complex semantics between entities in chain inference while pruning meaningless inference chains. Finally, we implement our method using semantic techniques, and two prototypes are implemented to show the semantic applications on the integration of tremendous heterogeneous healthcare knowledge. However, due to the lack of standard Chinese medical terminology, our results remain in relatively low accuracy. Our future work will focus on the improvements of those algorithms.

## Figures and Tables

**Table 1:** Detail information of metathesaurus of MKM.

Domain	Main sources	Number of entities
Bodily_part	Standard for Interface Technology of Health Data Sharing and Access: Part 1	79
Symptom/sign	Common Data Elements of Health Records (WS/T XXX-2009, CV5101.27, National Health and Family Planning Commission of China) manually collected from medical books	6809
Clinical_history	Classification and Coding for Value Domain of Health Data Element, 2012, National Health and Family Planning Commission of China (NHFPC), WS 364.4-2011, CV02.10.005	18
Laboratory_test	Medical Service Price Manual of Zhejiang	469
Disease/disorder	ICD9/10	20583
Drug	The Pharmacopoeia of People's Republic of China, 2015 Edition	526
Treatment_Method	Standards of Healthcare Information System Data Sharing and Interchanging of Wenzhou, 2013	9
Department	Standard for Interface Technology of Health Data Sharing and Access: Part 1	25

**Table 2:** Study relations.

Relations	Domain	Range	Seed facts
Located_in	Sign/symptom	Bodily_part	22
Cause	Clinical_manifestation	Diagnosis	22
Corresponded_to	Diagnosis	Treatment	20

**Table 3:** Input text corpus.

Genre	Documents	Sentences
OCRed medical book	663	11537
Open medical contents	2	24481

**Table 4:** Experiment results.

Relation	Harvest facts (per iteration)			Total harvest facts	Precision
	1	2	3		
Cause	379	3591	159	4129	45/50
Correspond_to	620	319	0	939	35/50
Located_in	106	289	28	523	36/50

**Table 5:** Evaluation of the contextual inference pruning algorithm.

Algorithm	Precision	Recall
Without pruning	50%	—
Naive Bayes	90%	91%
Logistic regression	92%	96%
SVM	91%	96%
ID3 decision tree	92%	91%
